# Acute Urinary Retention Leading to Post-renal Acute Kidney Injury Post-radiotherapy: A Case Report

**DOI:** 10.7759/cureus.116301

**Published:** 2026-09-15

**Authors:** Brayden Bitterman, Nitin Vaishampayan, Kimberly B Hart, Ramesh Boggula, Steven R Miller

**Affiliations:** 1 Department of Oncology, Wayne State University School of Medicine, Detroit, USA

**Keywords:** conformal radiation therapy, intensity-modulated radiation therapy (imrt), lower urinary tract symptoms, prostate cancer therapy, urinary obstruction

## Abstract

A man in his 70s with high-risk localized prostate adenocarcinoma underwent definitive external beam radiotherapy (EBRT) to the prostate and pelvis with concurrent androgen deprivation therapy using leuprolide. Eight days after completion of radiotherapy, he developed acute urinary retention (AUR) with overflow incontinence, constipation, and post-renal acute kidney injury (AKI), requiring hospitalization and bladder decompression with Foley catheter placement. Initial urine output was approximately 1 liter, and creatinine improved from 4.89 mg/dL to 1.46 mg/dL after decompression. Imaging demonstrated mild left hydronephrosis and chronic renal disease without an obstructing calculus. He was discharged home on tamsulosin and a bowel regimen with a Foley catheter in place. Persistent catheter dependence subsequently required transurethral resection of the prostate (TURP) with improvement in symptoms.

This case report describes a case of severe acute urinary retention with post-renal acute kidney injury occurring shortly after prostate and pelvic radiotherapy. The obstruction was likely multifactorial, with radiation-associated inflammation or edema potentially aggravating the underlying outlet issues and constipation. Prompt recognition and bladder decompression may permit rapid renal recovery and are important in patients with reduced renal reserves.

## Introduction

High-risk adenocarcinoma of the prostate is often treated with a combination of external beam radiotherapy (EBRT) to the prostate and regional lymphatics and long-term androgen deprivation therapy [1]. Recognizing the potential for pelvic EBRT to exacerbate urinary issues can help clinicians with the treatment of prostate cancer. Pelvic EBRT can exacerbate baseline lower urinary tract symptoms and precipitate acute urinary retention (AUR) via edema and inflammation of the bladder outlet and urethra [2]. When severe, this retention can lead to post-renal acute kidney injury, although severe retention is rare in published series [3-6]. The presence of pre-existing chronic renal disease can increase the likelihood that obstructive uropathy will develop into acute kidney injury (AKI). This can reduce the likelihood of short-term recovery after decompression and increase the risk of long-term progression to end-stage renal disease (ESRD) [7]. Thus, evidence of chronic underlying renal impairment warrants additional caution when treating these patients. Early recognition and prompt decompression are critical to reversing renal dysfunction and preventing permanent injury [8]. This case illustrates the intersection of oncologic therapy, urinary tract complications, and renal vulnerability in an older patient receiving guideline-concordant treatment for high-risk disease.

## Case presentation

A man in his 70s was noted to have an elevated prostate-specific antigen (PSA) of 16.6 ng/mL on routine blood work. He did not seek medical attention at that time, but a repeat PSA six months later was elevated at 24.02 ng/mL. He underwent an evaluation by his urologist, and a rectal examination at that time revealed no palpable nodules involving the prostate. A magnetic resonance imaging (MRI) of the pelvis revealed a prostate gland measuring 3.5 × 4.4 × 4.6 cm (volume: 38 cc) with no extraprostatic extension and no suspicious lymph nodes. A subsequent prostate biopsy revealed Gleason 4 + 3 = 7 disease, grade group 4. Additional staging studies, including a bone scan, revealed no scintigraphic evidence of osseous metastasis, and a computed tomography (CT) scan of the abdomen and pelvis demonstrated no evidence of metastatic disease.

His clinical stage per the American Joint Committee on Cancer (AJCC) tumor/node/metastasis (TNM) clinical staging system was cT1cN0M0, group III, with a prostate-specific antigen of 24.02 ng/mL, stage IIIA. He subsequently underwent a course of radiation and hormone therapy (leuprolide for 1.5-3 years), with hormone therapy starting at the initiation of radiation therapy. His initial International Prostate Symptom Score (IPSS) was 4 and was significant for nocturia two times at night and increased urinary frequency, urinating half the time in less than two hours.

Before the initiation of treatment, he underwent a planning session for radiation therapy. A CT scan was performed with the patient in the treatment position, and a cradle was fashioned for immobilization. A CT scan was obtained with 3-mm slices, and the images were imported into our treatment planning computer system to develop a radiation treatment plan. The clinical target volume (CTV) to receive 7000 centigray (cGy) (CTV7000) is defined as the volume that must be treated to achieve a cure. The CTV7000 consists of the prostate and the proximal 1 cm of the seminal vesicles. The planning target volume (PTV) to receive 7000 cGy (PTV7000) is the CTV7000 with a 5 mm expansion in all directions. The PTV accounts for uncertainties in planning or treatment delivery (Figure 1).

**Figure 1:**
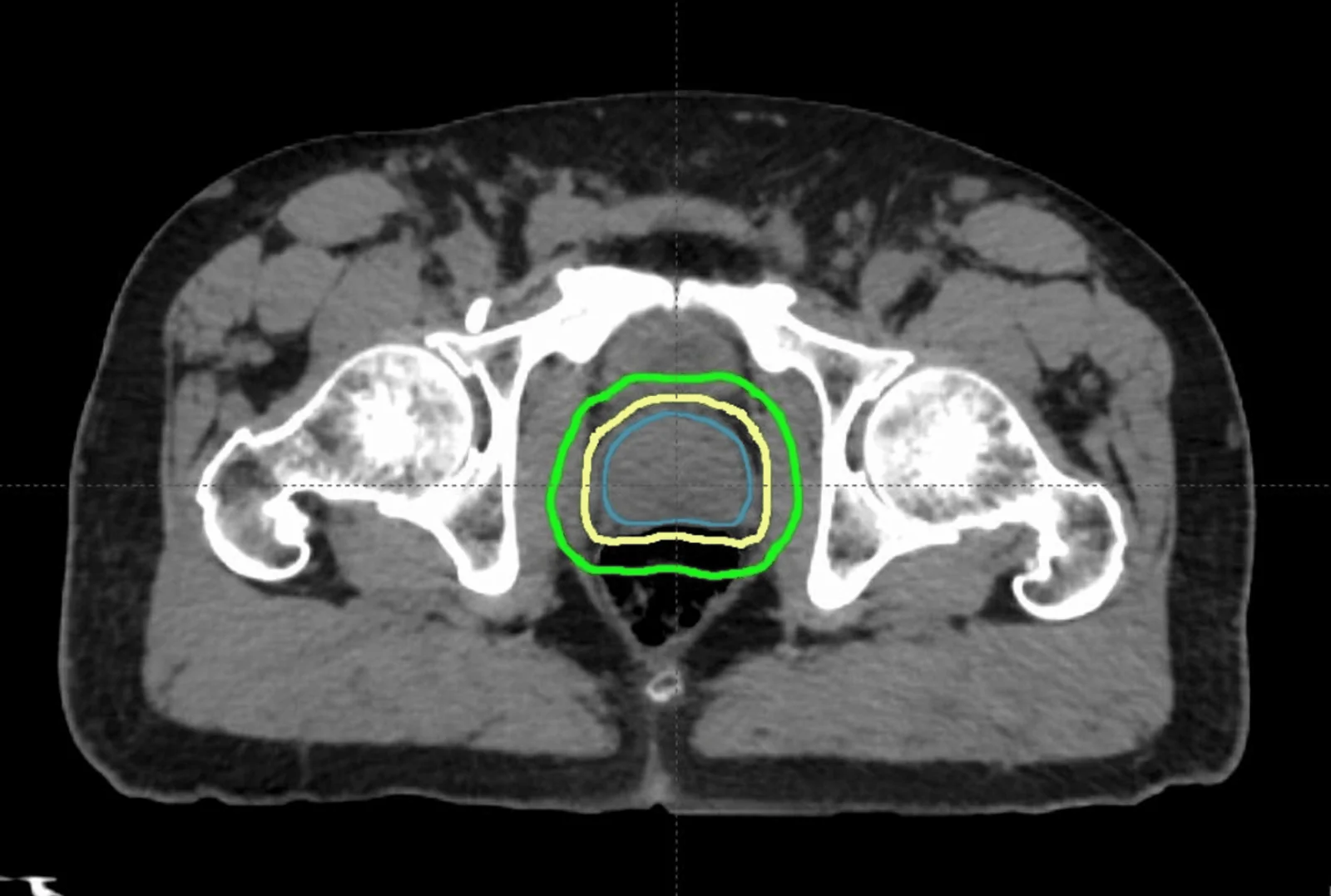
Dose distribution for treatment of the prostate using IMRT Light blue: CTV7000, yellow: 7000 cGy isodose line, green: 5000 cGy isodose line IMRT: intensity-modulated radiation therapy

The clinical target volume (CTV) to receive 5040 cGy (CTV5040), a radiation dose used to treat microscopic disease, included the internal and external iliac, common iliac, and obturator lymph nodes (pelvic lymph nodes). The PTV5040 cGy was a 7 mm expansion around this volume (Figure 2).

**Figure 2:**
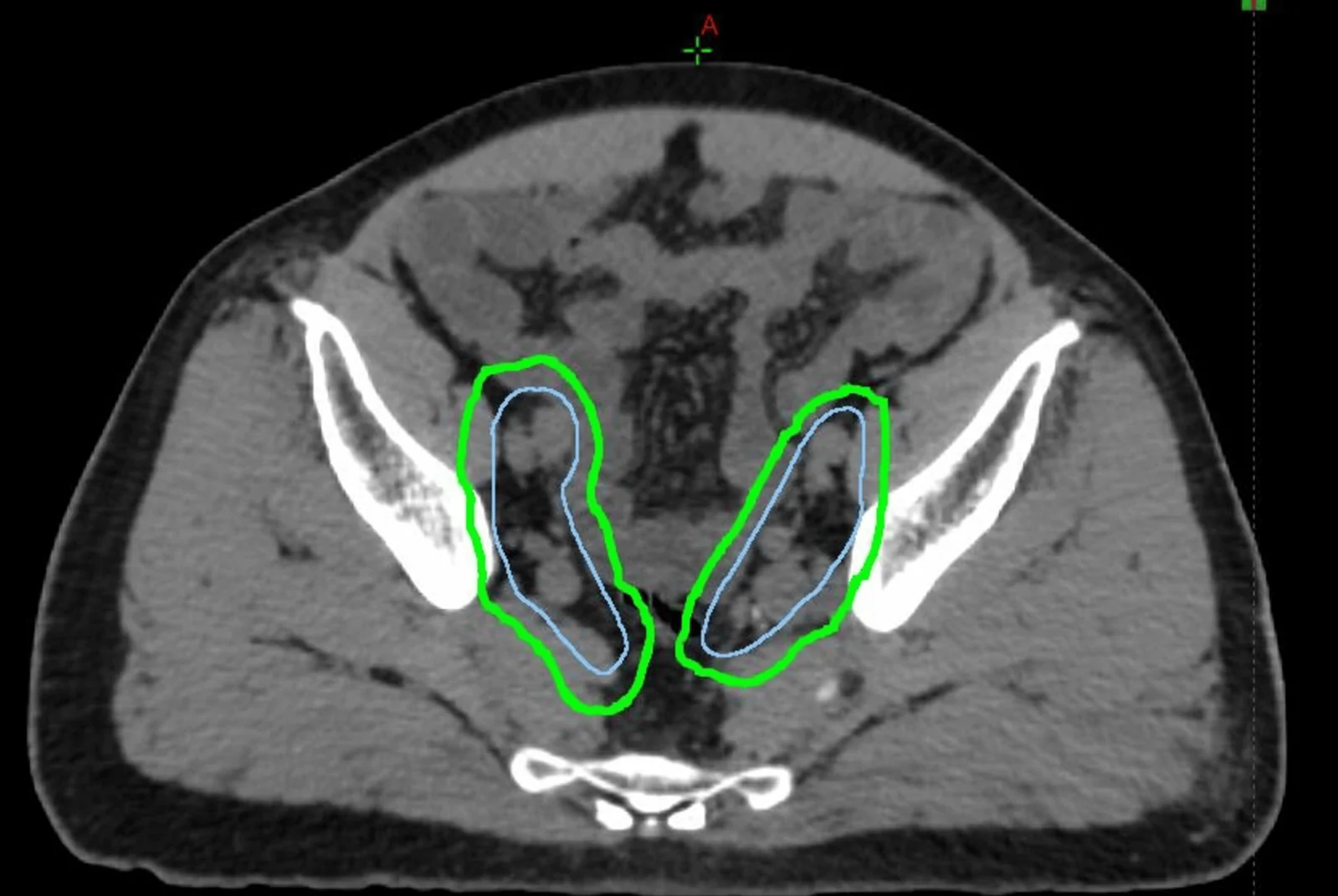
5040 cGy isodose line (green) and pelvic lymph nodes (light blue)

The patient received 7000 cGy to the prostate and proximal seminal vesicles in 28 fractions and 5040 cGy to the pelvic lymph nodes in 28 fractions using intensity-modulated radiation therapy (IMRT) with a simultaneous integrated boost (Figure 3 and Figure 4).

**Figure 3:**
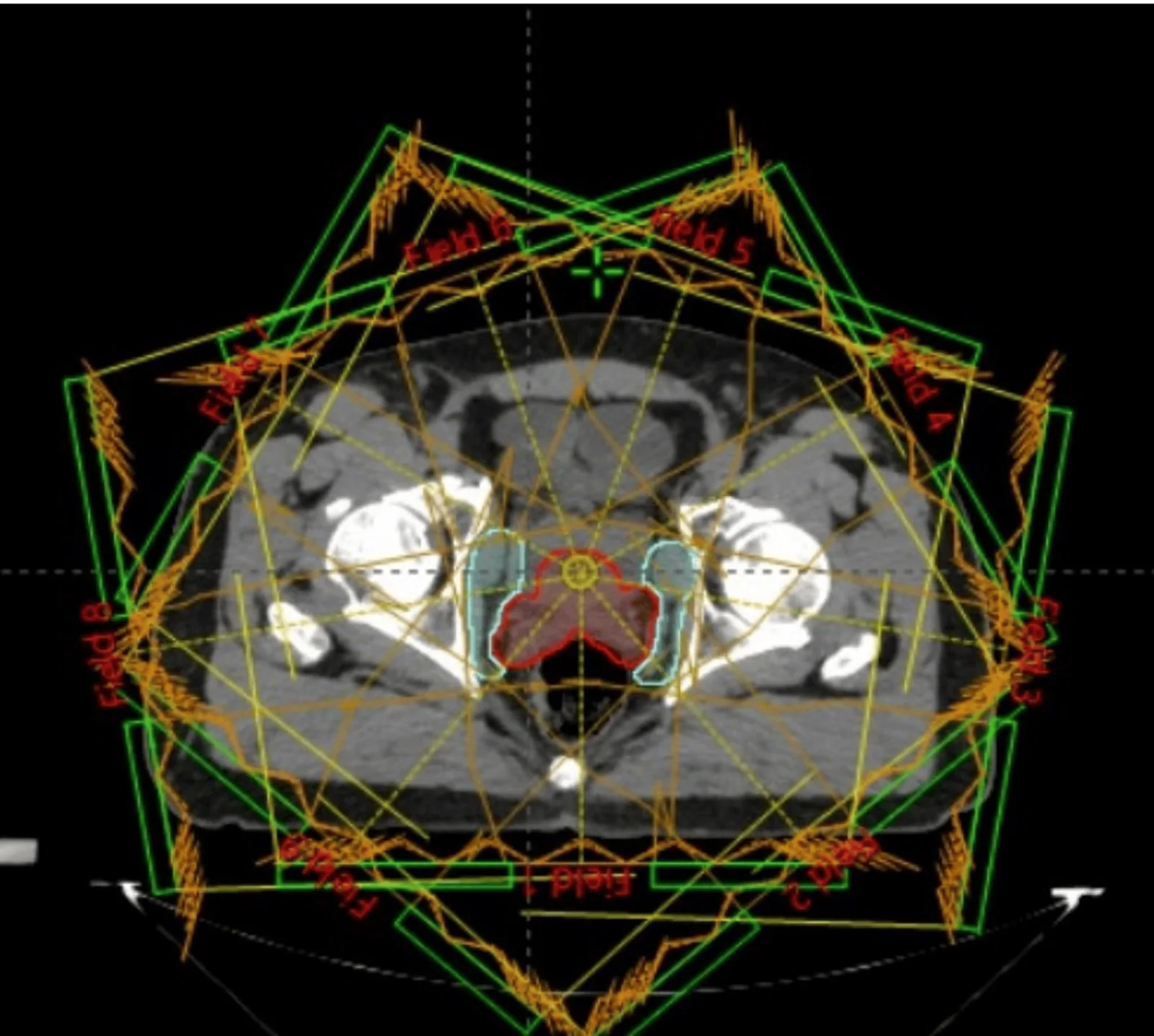
Radiation treatment to the prostate and pelvis using IMRT IMRT: intensity-modulated radiation therapy

**Figure 4:**
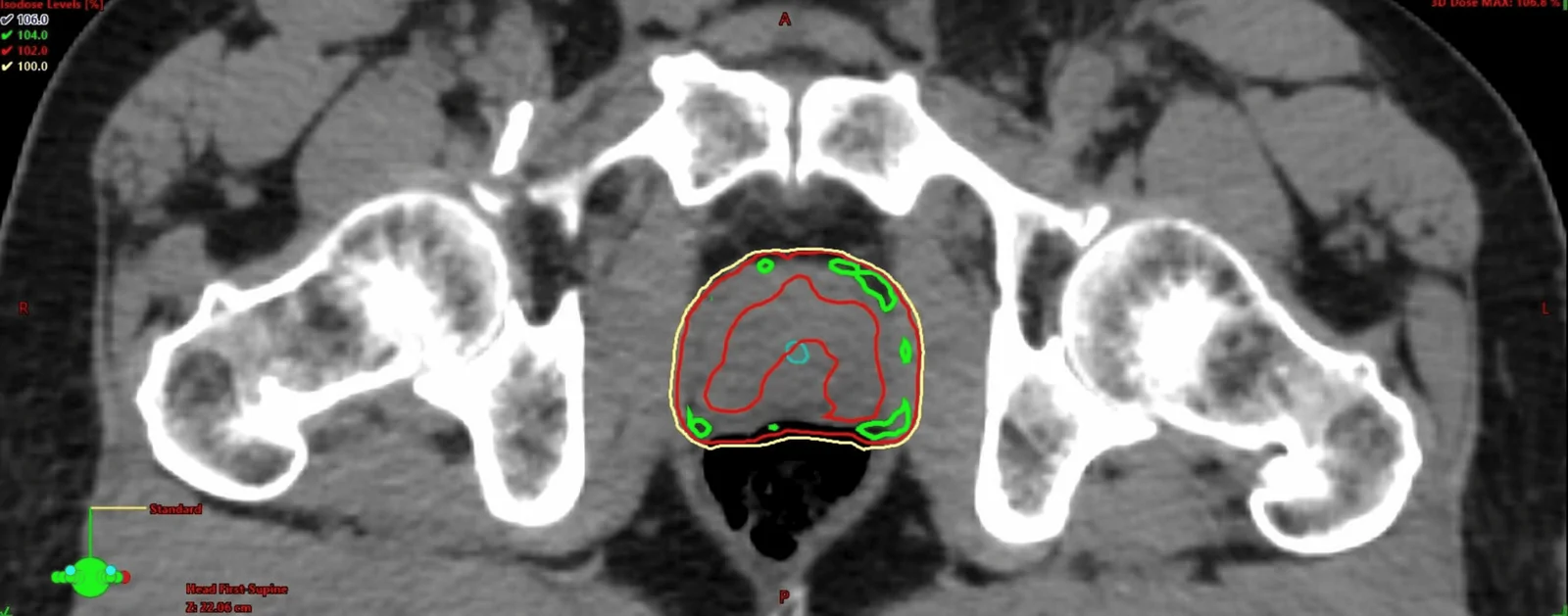
Isodose lines demonstrating dose to the urethra Light blue: urethra, yellow: 100% isodose line, green: 104% isodose line

A dose-volume histogram (DVH) displaying the dose to the bladder, rectum, and prostate is shown in Figure 5.

**Figure 5:**
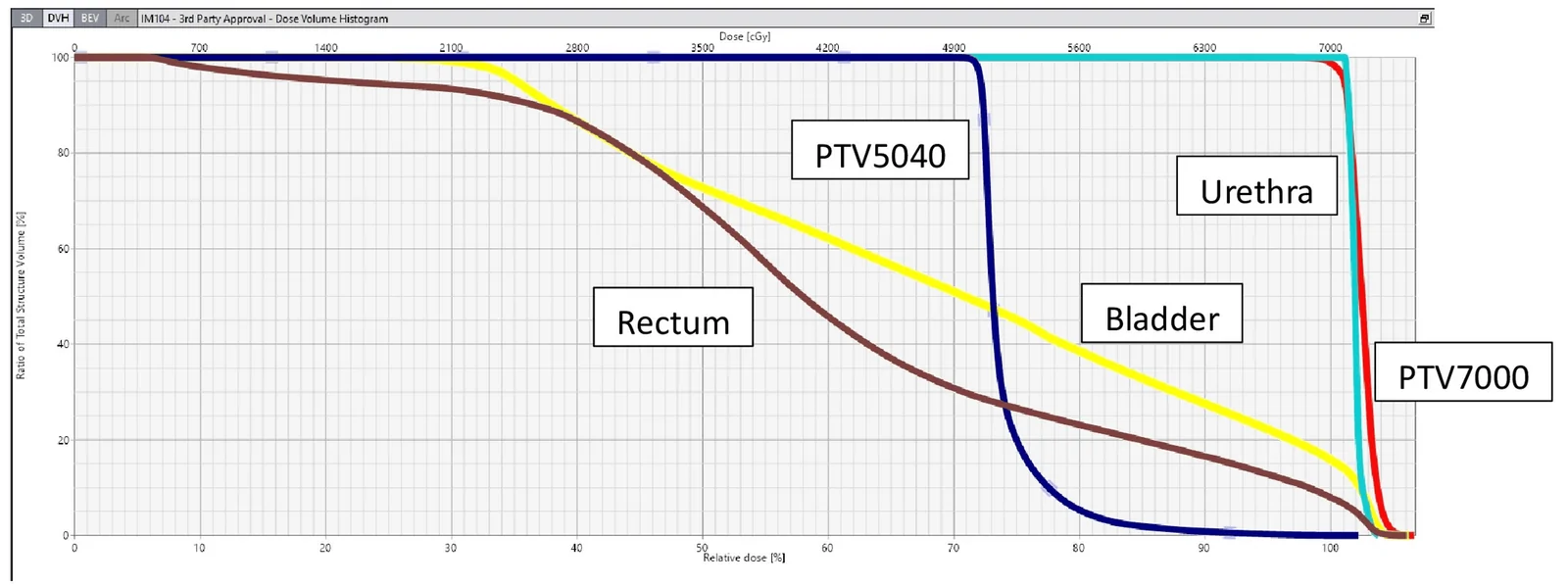
DVH displaying the dose to the rectum (brown), bladder (yellow), PTV5040 (dark blue), PTV7000 (red), and urethra (turquoise) DVH: dose-volume histogram

The bladder, rectal, and urethral constraints used for treatment planning are shown in Table 1. All dose constraints were met for this patient.

**Table 1:** Dose constraints for the bladder, rectum, and urethra QUANTEC: Quantitative Analysis of Normal Tissue Effects in the Clinic

Bladder		Constraint	Actual	Reference
D50%	<	6500 cGy	24.50%	NRG GU005 [9]
D35%	<	7000 cGy	15.80%	NRG GU005
D25%	<	7500 cGy	0.00%	QUANTEC [10]
D15%	<	8000 cGy	0.00%	QUANTEC
Rectum		Constraint	Actual	Reference
D50%	<	6000 cGy	19.40%	NRG GU005
D35%	<	6500 cGy	14.40%	NRG GU005
D25%	<	7000 cGy	8%	NRG GU005
D15%	<	7500 cGy	0.00%	NRG GU005
Urethra	<	Constraint	Actual	Reference
		8000 cGy, D0.1 cm3	0.00%	Flame [11]

The maximum dose to the bladder was 7437 cGy (left posterior bladder wall), with a mean dose of 4913 cGy. The maximum dose to the prostate was 7391 cGy, with a mean dose of 7154 cGy. Dose constraints for the rectum and bladder were as follows: D1cc rectum max 7700 cGy and D1cc bladder max 8000 cGy [12]. The dose to D1cc rectum was 7268 cGy and to D1cc bladder, 7242 cGy.

After completion of the treatment protocol, he developed acute urinary retention with overflow incontinence, associated constipation, and biochemical evidence of post-renal acute kidney injury with a creatinine of 4.89 mg/dL. He was subsequently hospitalized, and a Foley catheter was placed, with an initial urine output of approximately 1 liter. Afterward, his renal function improved, with a creatinine of 1.46 mg/dL. During the admission, he was treated with a bowel regimen leading to the return of bowel function. He was initiated on tamsulosin to optimize bladder outlet flow and a bowel regimen including polyethylene glycol to address constipation and reduce straining, which could exacerbate urinary symptoms.

A CT scan of the abdomen and pelvis without contrast obtained during admission demonstrated moderate left perinephric and periureteric soft tissue stranding with mild hydronephrosis and no obstructing calculus. A renal ultrasound performed the same day revealed chronic renal disease changes bilaterally without gross hydronephrosis. These findings supported a diagnosis of recent post-renal obstruction in a background of chronic parenchymal renal disease.

No surgical decompression beyond Foley catheterization was required during the initial episode, and there was no indication for emergent nephrostomy tube placement given the improvement in renal parameters and only mild hydronephrosis on imaging. Supportive care included monitoring renal function, maintaining hydration, and managing bowel habits to prevent recurrence of constipation-related strain. He was discharged with a Foley catheter in place and was initiated on tamsulosin and polyethylene glycol.

At a one-month follow-up visit, he reported that his bowel movements had normalized and that the Foley catheter remained in place pending urology follow-up for a trial of voiding and planned catheter removal. He described hot flashes consistent with ongoing leuprolide therapy. The prostate-specific antigen (PSA) level at that time was 0.833 ng/mL.

Two months after completion of radiation therapy, due to continued symptoms such as urinary retention with overflow incontinence, he underwent a transurethral resection of the prostate (TURP) with care taken not to resect excessively to prevent incontinence. His Foley catheter was removed approximately five days after the TURP with normal voiding. A PSA four months status post radiation therapy was 0.452 ng/mL.

## Discussion

Articles describing acute urinary retention resulting in post-renal acute kidney injury following pelvic radiotherapy are limited. Acute genitourinary toxicity is a common occurrence during or following pelvic radiotherapy [3-6]. Specific reports of severe acute urinary retention or obstruction requiring hospitalization and urethral catheterization are rare, particularly in the immediate post-treatment period. In a study by Tree et al. [3], one patient of 608 treated with moderately hypofractionated radiotherapy (6000 cGy over 20 fractions across a four-week course) developed grade 3 or greater urinary tract obstruction, which is defined by the Radiation Therapy Oncology Group (RTOG) as obstruction causing altered organ function (e.g., hydronephrosis or renal dysfunction) where invasive intervention is indicated [2]. Another study by Mandel et al., also using hypofractionated radiotherapy, reported one instance of grade 3 acute urinary retention, which required urethral catheterization, in 78 participants [4]. Additionally, while most large clinical trials studying radiation therapy toxicity report composite genitourinary toxicity scores without breaking down individual symptoms such as urinary obstruction, reports of acute grade 3 genitourinary toxicity are infrequent; the PACE-B trial reported only 6 cases in 432 patients [6].

During radiotherapy planning and delivery, the urethra, which lies within the target volume, is a dose-limiting structure. A clear dose-effect relationship exists between radiation delivered to the urethra and genitourinary toxicity. However, urethral delineation from the prostate can be difficult on CT scans; imaging techniques such as MRI or specialized contouring protocols can help. Addressing these challenges can optimize dose sparing while ensuring adequate tumor coverage [13].

When radiation-induced acute urinary retention fails conservative management with alpha-blockers and self-catheterization, surgical intervention with TURP becomes a logical option. However, the irradiated tissue makes surgery substantially more likely to result in incontinence (up to 18%-27% in reported series), possibly due to radiation-induced periurethral fibrosis, decreased bladder compliance, and damage to the proximal and distal sphincter mechanisms [5]. For this reason, conservative measures are preferred, and surgical intervention is avoided whenever feasible.

Differences in radiation fractionation schedules can influence toxicity outcomes. Large, randomized trials and meta-analyses revealed low rates of acute genitourinary toxicity among patients treated with conventional and moderately hypofractionated regimens [14]. In contrast, somewhat higher acute toxicity is associated with ultra-hypofractionated protocols [6,15].

Prospective prostate stereotactic body radiation therapy (SBRT) data have documented temporary catheterization for urinary retention and/or obstruction, including episodes occurring 6, 7, and 14 days after treatment [16]. An additional prospective study also reported Foley-requiring retention after therapy [17]. These two articles revealed that urinary retention alone may not be rare; the notable feature is severe obstruction with marked renal dysfunction.

Pelvic radiotherapy can precipitate or worsen lower urinary tract obstruction through mucosal edema, bladder neck inflammation, and exacerbation of pre-existing benign prostatic hyperplasia or subclinical outlet obstruction, particularly in older patients [2]. Evidence of chronic renal impairment, as seen in our patient, increases the likelihood that obstructive uropathy can present as acute kidney injury. This can reduce the likelihood of short-term recovery after bladder decompression and increases the risk of long-term progression to ESRD [7]. The additional presence of benign prostatic hyperplasia has been shown to increase the likelihood of post-radiation obstructive symptoms, underscoring the need for closer monitoring in this population [18].

Patients with acute urinary retention (AUR) present with an inability to void despite a distended bladder, discomfort, and exacerbation of symptoms with suprapubic palpation, as well as incomplete emptying and overflow symptoms [19]. Patients with symptoms of AUR should undergo prompt bladder decompression, with subsequent evaluation of the underlying cause. Clean intermittent catheterization plus alpha-blocker therapy is the treatment of choice for those with persistent symptoms [8]. Prompt bladder decompression with Foley catheterization leads to rapid improvement in renal function, emphasizing the reversible nature of post-renal acute kidney injury when addressed promptly, while imaging findings of mild hydronephrosis without calculi exclude competing structural causes such as ureteric stones.

From an oncologic standpoint, the selected radiotherapy regimen of 7000 cGy in 28 fractions to the prostate with pelvic nodal coverage to 5040 cGy, combined with 1.5-3 years of androgen deprivation therapy using leuprolide, is consistent with contemporary management strategies for high-risk localized prostate cancer [1]. Neoadjuvant androgen deprivation can significantly reduce prostate volume, which could potentially assist in decreasing the dose to the organs at risk. A study by Jethwa et al. demonstrated a prostate volume decrease of approximately one-third after almost five months of neoadjuvant hormone therapy, with total androgen suppression found to be more efficacious in maximizing cytoreduction than hormone therapy alone [20].

Our patient's prostate-specific antigen declined to 0.833 ng/mL and 0.452 ng/mL at one and five months after completing radiation therapy [21], although durable biochemical control will require longer-term assessment. The patient also maintained a great functional status despite treatment-related toxicity, suggesting that with resolution of catheter dependence and continued surveillance, functional status may remain acceptable [22].

This case underscores the importance for radiation oncologists, urologists, and primary care clinicians to anticipate obstructive urinary complications and renal sequelae in patients with high-risk prostate cancer, institute early decompression and supportive measures, and coordinate follow-up across disciplines to optimize both oncologic and functional outcomes.

## Conclusions

Severe acute urinary retention with post-renal acute kidney injury can occur shortly after definitive prostate and pelvic radiotherapy. This is especially true in patients with underlying chronic renal disease or benign prostatic hyperplasia. In these patients, substantial improvement in renal function with Foley catheter decompression supports a clinically significant obstructive component. Causes of the obstruction are most likely multifactorial, with radiation-associated inflammation or edema potentially exacerbating the symptoms.

Clinicians should maintain a low threshold for evaluating new urinary obstructive symptoms during and shortly after undergoing prostate radiation therapy, particularly in patients with baseline bladder outlet obstruction and/or evidence of pre-existing chronic renal impairment.
